# Editorial for Brian Sciences Special Issue “Epidemiology of ASD Services: Unmet Need, Barriers and Innovative Solutions”

**DOI:** 10.3390/brainsci12070895

**Published:** 2022-07-08

**Authors:** Aldina Venerosi, Flavia Chiarotti

**Affiliations:** Center for Behavioral Sciences and Mental Health, Istituto Superiore di Sanità, Viale Regina Elena 299, 00161 Roma, Italy; flavia.chiarotti@iss.it

We are very honoured by the collaboration we had with the editorial staff of *Brain Sciences* for the realization of the special issue “Epidemiology of ASD Services: Unmet Need, Barriers and Innovative Solutions”.

The articles collected in the Special Issue aim to emphasize the need for investing an increasing number of competencies for the implementation of programs dedicated to the management of care for people with autism. One of the priorities recognised internationally is to improve the tailored health approach. The spectrum of behavioural manifestations and the heterogeneity of other symptoms, such as those in neuropsychological, neurological, and general physical areas, have raised the awareness that it is necessary to build care paths characterized by a strong multidisciplinarity and capacity of the team. These two characteristics represent important assets to address the complexity that autism imposes on the growing individuals and their adult life. The ability to analyse how the various phenomenological aspects interact with each other can make a real difference to increase the effectiveness of interventions. Moreover, a management that takes into account the multiple aspects of autism will be more able to respond to the needs of care of the individual. To achieve this goal, it is necessary to make a large investment in many fields of research and the ability of governments to use the results of this research to implement services that are supportive to people with autism at all ages of their life. The selection of studies presented in the Special Issue gives an example of the disciplinary heterogeneity involved in the research, which is useful to respond to the needs of people with autism.

Monitoring the processes adopted by the mental health services for childhood and adolescence to manage autism represents a source of irreplaceable information. Monitoring the process can also provide epidemiological data about the population diagnosed with autism that has access to these specialised services. Although this type of analysis may not meet the methodological criteria of a rigorous epidemiological study, it represents an ecological observational study that can highlight the critical issues that clinicians have to cope with. One factor that emerges from this type of analysis is the need to introduce a model of recording clinical patient information, including milestone of his/her care pathway. This model should be as close as possible to a reference model or a specific standard of quality extracted from the best available scientific evidence. The study “Management of Autism Spectrum Disorder in Italian Units of Child and Adolescent Mental Health: Diagnostic and Referral Pathways” demonstrates that the individual clinical charts currently adopted can fail to report complete data, making it difficult to monitor the clinical processes followed by each patient, but also the individual symptomatological picture and its evolution. It is possible that the clinicians have a clear idea of the specific patient’s characteristics, but they do not systematically report the information in the patients’ medical records, compromising communication between professionals, and even the historical memory of that case. Moreover, the study shows that starting from the analysis of medical records, it is possible to highlight the delays in the implementation of clinical recommendations, including the adoption of specific interventions. The adoption of a model of reporting, preferentially digital, will allow a better assessment of the evolution of the individual autistic disorder, and then the formulation of hypotheses of intervention or modifications of intervention. At the same time, a systematic and digital collection based on local health services allows the monitoring of the ability to implement the main scientific recommendations for autism management, and, therefore, allows you to verify the need for investment in human and instrumental resources. Finally, a complete and rigorous case registration can contribute to the epidemiological knowledge of the disorder.

Early ASD diagnosis is considered a foremost goal endorsed by many government programs worldwide. The core behavioural features of ASD emerge within the first two years of life, but the clinical diagnosis is rarely attained before the third birthday. To empower primary care to recognise early ASD signs, standardised screening tools were developed. These scales can be routinely used by paediatricians during the 18 and 24 month-scheduled well-visits. The screening is a time-consuming process that should be available widely across the country to be effective, thus dedicated government programmed resources are needed. Resource-poor country areas and/or geographically difficult areas need innovative screening models able to support the universal access to screening and consequently facilitate early ASD diagnosis. Findings suggest that there is potential for telehealth and e-health models to improve access to assessment and diagnosis of ASD. The study “Integrating a New Online Platform in Primary Care for Early Detection, Referral, and Intervention in Autism Spectrum Disorder: The First Italian Pivotal Project” is a pilot implementation of the first Italian online web-based screening tool by the use of an innovative web app (WIN4ASD) for paediatricians. The study adds to the recent literature that supports telehealth by the use of web-based platform to improve the access to assessment and diagnosis of ASD. Furthermore, the study focused on the enhancing of the continuity of care in the field of neurodevelopmental disorders, specifically for ASD, by employing digital e-health tools connecting general practitioners with child neuropsychiatry specialists. Though this field of research is in the very early stages and includes studies with small sample size, the findings suggest that this kind of solution is promising.

Digital technologies also offer unique possibilities for innovative approaches to ASD intervention. In particular, multimedia technologies and virtual reality can be used to manipulate sensory, motor, interpersonal, and cognitive processes contributing to both disentangle and intervene on specific functions. Developmental cognitive neuroscience provides new insights on both typical and atypical neurobehavioral functioning. Multi-sensory channels and their integration with the evolution of motor skills during development builds up the bodily self-perception and the multiform environment aspects awareness. To help multidimensional development potentially target the ASD core symptoms, specific multimedia and virtual reality activities can be designed. These activities are able to stimulate bodily-self experiences, crucial for building up a coherent sense of self and lay the foundation for interacting with the external world. Atypical bodily self is an early marker of heterogeneous neurodevelopmental conditions (such as ASD) and seems to be under-targeted in research and clinical approaches. The study “Multimedia Interventions for Neurodiversity: Leveraging Insights from Developmental Cognitive Neuroscience to Build an Innovative Practice” outlines, in the framework of neuroconstructivism, the main principles that should be taken into account to shape multi-technologies activities and provides some practical examples to demonstrate the feasibility of this intervention in the clinical practice. As the authors stated, the generalisation of these innovative approaches to ASD intervention needs to be supported by further studies that establish and test the optimal timing, frequency, and duration of these interventions and evaluate the effects of these simulations on children’s sensory, motor, cognitive, and social development, as well as assessing their applicability to daily life contexts and durability over time. Furthermore, collecting neural measures before, during, and after an innovative training that uses digital technologies will be crucial to deepen how the bodily self is expressed according to the various stimulations and becomes particularly informative for clinicians to translate new knowledge in clinical practices. Finally, the use of multimedia technologies needs the introduction of new competencies in the clinical setting requiring both psychological and technical expertise.

Steering the health services to promote the care of people with ASD consistently with the new advances in the field of ASD pathogenic processes is a priority to build a tailored response to the needs of these patients. Recent biological approaches to ASD clinical intervention emphasises the importance to consider ASD as a developmental multisystem disorder that involves the complex interaction among different districts of the organism, besides the brain. Thus, the embryo–foetal period appears as the crucial window of opportunity for applying preventive strategies to support neurodevelopment. The woman’s health in pregnancy should be a priority to preserve adaptive molecular pathways beginning in utero and the inter talking between the immune response, the oxidative stress/mitochondrial dysfunction, and the neural maturation and functioning. Preventing the dysbiosis impact on neurodevelopment and brain functioning across the lifespan could decrease health loss and the higher mortality in ASD. The review “Dynamic and Systemic Perspective in Autism Spectrum Disorders: A Change of Gaze in Research Opens to A New Landscape of Needs and Solutions” gives a synthesis of the biological evidence underlying the complexity of the pathogenic model of ASD, detailing the broad array of metabolic, immunologic, and microbiological imbalances affecting ASD people, from pregnancy throughout their entire lifespan. These data support a multisystem pathogenic view overcoming the apparent conflict between biological perspective and the psychosocial approach, which limits the current conceptualisation of ASD. The conceptual framework that emerges from the analysis of these data is that the biological complexity of ASD and its implications for health require the enhancement of clinical skills on these topics, to achieve an effective multi-disciplinary healthcare model aimed at an integrated treatment approach that takes into account psycho-behavioural, social, and physical aspects. Well-balanced training courses could be a promising starting point to make the necessary change.

The complexity of the ASD condition includes the high impact that this developmental disorder has on the whole family. The gap between children’s needs and their satisfaction, with special regard to the shortage of social and healthcare services, is a source of burden, particularly after the transition to adulthood. Evidence indicates that the lack of services and support to the family affects their health as well as their quality of life. The study “The Impact of Health and Social Services on the Quality of Life in Families of Adults with Autism Spectrum Disorder (ASD): A Focus Group Study” aimed to gather a comprehensive view on how parents of adults with ASD perceive (and interact with) health and social services. The goal of this focus group study was to identify specific areas of change useful to influence autism intervention strategies so that they more effectively meet the needs of young people with autism and their families. The quality of life conceptual framework guided data collection and analysis as part of a directed theory-driven content analysis approach. By means of this narrative approach, the study outlines more of the lack of the structured care pathways and the low level of integration of the different services. During the focus group, parents argued for a greater role of the institutions in order to facilitate the building of networks that are really inclusive for persons with autism in society and to support the implementation of innovative solutions for the welfare system. Furthermore, parents stressed the need for the provision of support to the family. An increasing number of experiences sees parents as self-directed agents of solution for the management and individual accomplishment of their children in the community in which they live. This experience represents a useful indication for re-thinking the approach of the main social and health care agencies, which are required to drop the prevailing prescriptive behaviour and endorse a participatory one.

Interestingly, the model outlined in the article “Project Extension for Community Health Outcomes (ECHO) Autism: A Successful Model to Increase Capacity in Community-Based Care” presents an innovative strategy to improve local health and ASD community for addressing the complex needs of children with ASD and reducing disparities often present in rural and underserved communities. The ECHO model follows a “hub and spoke” design, where an interdisciplinary “hub team” of content experts provides guided practice to “spokes”, or professionals in local communities, to build capacity. This model was applied with over 400 replication sites globally for different conditions (HIV, diabetes, chronic pain, endocrinology, addiction, behavioural health disorders, and psychiatric conditions) and it is now adapted to provide evidence-based care to children with ASD and their families. The first implementation of the ECHO program for ASD was in Missouri and focused on the empowerment of general practitioners in order to detect ASD and manage the co-occurring conditions (ECHO Autism STAT program). Data collected demonstrated two main effects of this program: (i) accelerating early access to autism diagnosis; (ii) reducing the burden of travel for families by an average of 173 miles. A further innovative aspect of this model implemented by the Missouri ECHO Autism Primary Care program was to include a parent of a person with ASD and an autistic person as a content expert on the hub team. This reflects the adherence of the Autism ECHO model to the principle of patient- and family-centred care with the adoption of shared decision making. The ECHO experience reported in this article encourages the introduction of new models of care that are close to the people to be cared for, that take into account the point of view of the persons with ASD and their family, and that develop multidisciplinary and integrated competencies to cope with the complex needs of person with ASD. Research is clearly needed, preferably by means of randomized control trials (RCTs), to evaluate the effectiveness of these programs to improve functionality and clinical outcomes in autism spectrum disorders.

To conclude, the contributions that are part of the Special Issue “Epidemiology of ASD Services: Unmet Need, Barriers and Innovative Solutions” published by *Brain Sciences* emphasize that many different aspects must be taken into account when you want to offer support to the needs of people with ASD. New technologies, together with a new idea of service organization that recognises people with ASD and their families part of the decisions that affect them directly, outline areas of progress and ongoing challenges, which in the coming years should be the objective of high quality research to give us more certain data. A particular thank you to all authors who submitted their work to this Special Issue and to the reviewers for dedicating their time and for helping to improve the quality of the published manuscripts. In conclusion, we hope that this contribution to the ASD literature will increase research interest for the specific needs of the persons with ASD and their families, as well as for the operators who in various ways work with them. Indeed, data from research may innovate the provided service to become more and more tailored to the individual needs showed by each person with ASD.

